# Aix-Les-Bains

**Published:** 1890-05-17

**Authors:** 


### Aix-les-Bains.

It is gratifying as a proof of the satisfactory state of Her Majesty's health that she should have chosen to go to Aix-les-Bains so early in the season as April. The great majority of the visitors to that favoured spot do not venture there until later, with the consequence that hotels are partly closed, partly whitewashing and redecorating, partly with a dining-room and a few bed-rooms open to the public while the entertainment rooms are cleaning and the furniture piled away in a corner. The climate of Savoy and Dauphine is very much milder than that of Lucerne and Baden. Nevertheless, on emerging from the Mont Cenis Tunnel there is snow lying upon peaks of the French alps, which in respect rival Mont Blanc in elevation. And so to Aix. The little station stands at the foot of the town, which rises street by street to the Hotel Splendide, the baths, post-office, and bank, while still higher follows a succession of fine hotels and villas. Without the picturesque Lake of Chambéry, stretching away as it does for miles, the Spa would lose much; but even then the lofty mountains, spurs of the loftier Alps, would render its scenery grand and the air bracing.

Art has supplied every convenience and luxury. There is the Casino and its grounds locked against the intruder, while the victims of the coming summer are where?—who can say. The hotels are magnificent, at the same time impressing one with a sense of refinement. It is not so easy to find a place to dine, for the painters are busy here, and one has to retire after reading the concert programme of some weeks hence. Perseverance, however, is rewarded, and a hotel is found with table d'hôte at six francs—five shillings. Dining à la carte is far more expensive, however *récherché* the sole with the flesh delicately separated from the bones by the waiter before offering it to his guest. It is the Englishman's way to read his paper over his cover unless he is accompanied by his wife. The *Times* is missing, but the *New York Herald*, *Galignani's Messenger*, and the various journals specially printed to suit the Anglo-American tourist are at hand.

A single large establishment contains the baths in their many varieties and the medicinal waters flowing from three taps upstairs. Then there is a list of the local physicians hanging in the hall, one bearing an unmistakably Scotch patronymic. He has not, however, attained the distinction of Sir A. Garrod, with an avenue in the town named after him. The bath-rooms are excellent, the floors of slabs of polished marble heated so as to be very grateful to the feet, and the cost is 1s. 6d. Out of deference to her Majesty, an English shilling passes current for the value of a franc. The principal waters drunk are the alum water and the sulphur water, both hot, of a temperature of from about 108 deg. to 115 deg.

Charges in Aix are high, for it is a resort, upon the one hand, of the wealthy, and, upon the other, of the spendthrift. Accommodation may, however, be had in small, unpretentious boarding-houses for half-a-crown a night. There are drawbacks. John Bull, after a long night journey and a short rest, gets up, and orders his favourite breakfast—bacon and eggs—and he adds, "Let me have it à l'Anglaise." Travelling has given him appetite, and he is impatient for his meal, when his French hostess brings him a couple of eggs too underdone to eat and a couple of slices of cold boiled ham. What use in complaining; perhaps, besides, his knowledge of French will not carry him very far in that direction.

